# MutationalPatterns: comprehensive genome-wide analysis of mutational processes

**DOI:** 10.1186/s13073-018-0539-0

**Published:** 2018-04-25

**Authors:** Francis Blokzijl, Roel Janssen, Ruben van Boxtel, Edwin Cuppen

**Affiliations:** 1 Center for Molecular Medicine and Oncode Institute, University Medical Center Utrecht, Utrecht University, Universiteitsweg 100, 3584 CG Utrecht, The Netherlands; 2 Princess Máxima Center for Pediatric Oncology, Heidelberglaan 25, 3584 CS Utrecht, The Netherlands

**Keywords:** R, Base substitutions, Somatic mutations, Mutational signatures, Mutational processes, Transcriptional strand bias, Replicative strand bias

## Abstract

**Background:**

Base substitution catalogues represent historical records of mutational processes that have been active in a cell. Such processes can be distinguished by various characteristics, like mutation type, sequence context, transcriptional and replicative strand bias, genomic distribution and association with (epi)-genomic features.

**Results:**

We have created MutationalPatterns, an R/Bioconductor package that allows researchers to characterize a broad range of patterns in base substitution catalogues to dissect the underlying molecular mechanisms. Furthermore, it offers an efficient method to quantify the contribution of known mutational signatures within single samples. This analysis can be used to determine whether certain DNA repair mechanisms are perturbed and to further characterize the processes underlying known mutational signatures.

**Conclusions:**

MutationalPatterns allows for easy characterization and visualization of mutational patterns. These analyses willsupport fundamental research into mutational mechanisms and may ultimately improve cancer diagnosis and treatment strategies. MutationalPatterns is freely available at http://bioconductor.org/packages/MutationalPatterns.

**Electronic supplementary material:**

The online version of this article (10.1186/s13073-018-0539-0) contains supplementary material, which is available to authorized users.

## Background

The genomic integrity of cells is constantly challenged by both endogenous and exogenous sources of DNA damage, such as ultraviolet (UV) light and spontaneous reactions. Cells harbour a collection of DNA repair mechanisms to counteract these assaults. Not all lesions are, however, correctly repaired prior to replication, resulting in mutation incorporation into the genome [1]. Acquired mutations can have functional consequences and contribute to the development of diseases such as cancer and accelerate aging [2, 3]. Knowledge on the causative mutational processes is therefore important for understanding disease aetiology and could be valuable for future development of therapeutic strategies aimed at preventing or treating disease [4].

Each mutational process is thought to leave its own characteristic mark on the genome. For example, AID/APOBEC activity can specifically cause C > T and C > G substitutions at TpCpA and TpCpT sites (of which the underlined nucleotide is mutated) [5]. Thus, patterns of somatic mutations can serve as readout of the mutational processes that have been active and as proxies for the molecular perturbations in a tumour [6]. In the past few years, large-scale analyses of human tumour genome data across different cancer types have revealed 30 recurrent base substitution patterns, which are archived in the Catalogue of Somatic Mutations in Cancer (COSMIC) (http://cancer.sanger.ac.uk/cosmic/signatures). These “mutational signatures” are characterized by a specific contribution of 96 base substitution types with a certain sequence context [5]. Some mutational signatures could be linked to specific biological processes through association with exposure to carcinogens, such as tobacco smoke [6, 7], or the deficiency of DNA repair processes, such as nucleotide excision repair (NER) [8]. However, since multiple processes are typically disrupted in tumours, it is difficult to directly link a specific DNA repair and/or damage process to a signature based on genomic analyses of tumours. As a result, the aetiologies of most mutational signatures that were computationally derived from human tumour data are currently unknown [5]. In order to fully exploit mutational signature analysis for cancer diagnosis and treatment decision, the underlying molecular mechanisms need to be revealed.

Recent advances in gene editing have enabled researchers to knock out specific DNA repair mechanisms and directly evaluate the effect on patterns of mutation accumulation [9]. For example, human adult stem cells in which the base excision repair (BER) protein NTHL1 was deleted using clustered regularly interspaced palindromic repeats (CRISPR)/CRISPR-associated proteins (Cas9) genome editing, showed a predominant increase of “signature 30” mutations [10], for which the underlying molecular mechanism was previously unknown. In a similar fashion, mutational signatures can be linked to specific sources of mutagenic stress, by studying their contribution in cells that are exposed to a specific carcinogen. To link DNA damage or repair to previously known mutational signatures, it is essential to have a method for the quantification of mutational signature activity in newly generated mutation catalogues.

In addition to mutational signatures, mutational strand asymmetries provide meaningful information on the underlying mutational processes. For example, transcriptional strand asymmetry arises in expressed genes through increased transcription-coupled NER (TC-NER) on the transcribed strand and/or increased damage on the exposed untranscribed strand [11]. Decrease of this asymmetry potentially reveals a deficiency of TC-NER. Furthermore, replicative strand asymmetry can arise as a result of the different DNA polymerases that are used for the replication of the leading and lagging strands, which have distinct fidelities [11]. Increased replicative asymmetry may serve as a proxy for reduced proofreading capacities of polymerase ε (POLE) at the leading strand [12], or dysfunctional mismatch repair (MMR), which normally repairs most DNA polymerase mistakes [10, 11].

The distribution of mutations across the genome also provides valuable clues on the mutational mechanisms. For example, exposure to UV light and alcohol increases the activity of error-prone DNA repair, involving translesion polymerase η (POLH), specifically at H3K36me3 chromatin in various cancer types. However, this effect does not affect the overall mutation rate or spectrum. Rather, the carcinogenic effect might be a result of the differential targeting of mutations towards active genes, which are more likely to be consequential [13]. Analysis of the regional mutation rates in expressed genes and/or H3K36me3-associated regions is thus important for revealing this specific mutational mechanism. Finally, the distance between consecutive mutations can be evaluated to identify the clustered mutagenesis called “kataegis”, a phenomenon associated with APOBEC overactivity [14], which has been shown to correlate with low responses to tamoxifen [15, 16].

Different mutational characteristics, such as type, sequence context, genomic distribution, association with genomic regions and transcriptional and replicative strand bias, are collectively meaningful for the dissection of the molecular mechanisms underlying mutation accumulation. Assessment and visualization of this wide variety of mutational patterns is, however, a challenging task. Here, we describe MutationalPatterns, an R/Bioconductor package that makes these diverse mutational pattern analyses available to a broad range of researchers. In addition, we provide a very efficient method to determine the contribution of known (e.g. COSMIC) or user-specified mutational signatures in individual samples. Using this method, it is possible to (1) estimate the contribution of known signatures in cells with (experimentally) altered DNA repair or damage and (2) evaluate the activity of signatures in individual tumours. With these functionalities, MutationalPatterns is a versatile software package that facilitates the study of mutagenic agents and processes, the molecular dissection of existing mutational signatures and the identification of molecular defects in individual tumours to improve diagnosis and treatment decision.

## Implementation

We implemented MutationalPatterns within the R/BioConductor platform [17], which is a widely used open-source software project for computational biology and bioinformatics. This platform provides easy integration with other R/BioConductor packages and workflows. All visualizations are generated with the powerful data-visualization package *ggplot2* [18], which can easily be adjusted to individual requirements with additional ggplot2 commands. Moreover, publicly available genomic datasets can be retrieved using the *BiomaRt* package [19] and used in the analyses, which allows exploration of a vast source of genomic annotation data from popular sources such as Ensembl (www.ensembl.org). In addition, in-house or publicly available experimental data, such as RNA-seq and chromatin immunoprecipitation sequencing (ChIP-seq) data, can be integrated.

### Data import and mutation types

**Fig. 1:**
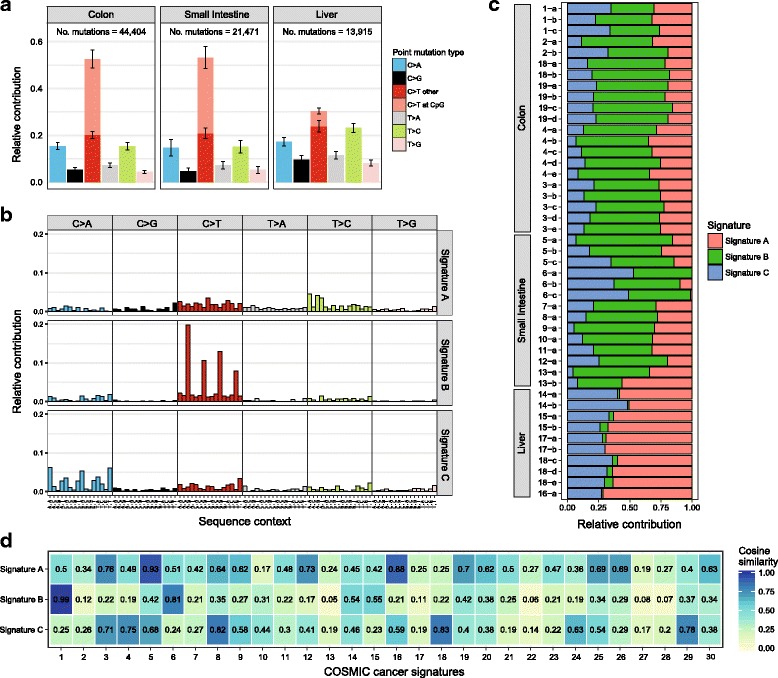
Characteristics of somatic mutations acquired in human ASCs of different tissues. **a** Relative contribution of the indicated mutation types to the point mutation spectrum for each tissue type. *Bars* depict the mean relative contribution of each mutation type over all ASCs per tissue type and *error bars* indicate the standard deviation. The total number of somatic point mutations per tissue is indicated. **b** Relative contribution of each indicated trinucleotide change to the three mutational signatures that were identified by NMF analysis of the somatic mutation catalogues of the ASCs. **c** Relative contribution of each mutational signature for each sample. **d** Heatmap showing the cosine similarity of the mutational signatures in **b** with the COSMIC signatures

**Fig. 2:**
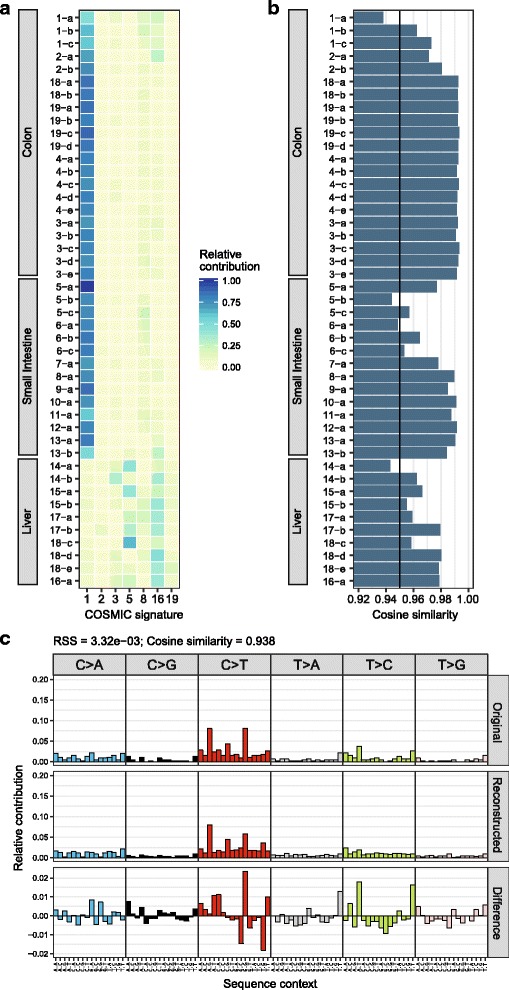
Reconstruction of mutational profiles using known mutational signatures. **a** The optimal relative contribution of COSMIC signatures to reconstruct the mutational profiles of the samples. The signatures with at least 10% contribution in at least one of the samples are plotted. **b** Cosine similarity between the original mutational profile and the reconstructed mutational profile based on the optimal linear combination of all 30 COSMIC signatures. The *line* indicates the threshold of cosine similarity = 0.95. **c** Relative contribution of each of the 96 trinucleotide changes to the original mutational profile (*upper panel*) and the reconstructed mutational profile (*middle panel*), and the difference between these profiles (*lower panel*) for the ASC with the lowest cosine similarity (*1-a*). The residual sum of squares (RSS) and the cosine similarity between the original and the reconstructed mutational profile are indicated

Any set of base substitution calls, can be imported from a Variant Call Format (VCF) file and is represented as a *GRanges* object [20], a widely used data structure that allows very efficient computations including subsetting and overlapping with other genomic regions. MutationalPatterns reads VCF files in parallel, which reduces the time from *O(n)* to *O(n/c)*, where *n* is the number of VCF files, and *c* the number of cores available. All available reference genomes can be installed with the *BSGenome* package (http://bioconductor.org/packages/BSgenome/). Once the data are imported, the sequence context of the base substitutions can be retrieved from the corresponding reference genome to construct a mutation matrix with counts for all 96 trinucleotide changes using “mut_matrix”. Subsequently, the 6 base substitution type spectrum can be plotted with “plot_spectrum”, which can be divided per sample group, such as tissue type (Fig. 1a). Error bars indicate the standard deviation over the samples per group. For the C > T base substitutions, a distinction can be made between C > T at CpG sites and C > T at other sites, as deamination of methylated cytosines at CpG sites is a frequently active mutational process [5]. Moreover, a barplot with the 96 trinucleotide changes can be generated for each sample with “plot_96_profile”. At least 200 mutations are typically required to construct a representative mutational profile. Differences between two mutational profiles can be visualized using “plot_compare_profiles” (Fig. 2c), in which the residual sum of squares (RSS) and cosine similarity values are indicated.

### Mutational signatures

Mutational signatures can be extracted *de novo* from the mutation count matrix, which contains counts of all 96 trinucleotide changes in each sample, using non-negative matrix factorization (NMF) with “extract_signatures”. For this dimension reduction approach, the number of signatures is typically small compared to the number of samples in the mutation matrix. MutationalPatterns uses the implementation of R package *NMF* [21], which can also be used to estimate the optimal number of different mutational signatures that can be extracted from the data. Alternatively, novel probabilistic methods for identifying mutational signatures [22, 23] can be used to extract signatures *de novo*, and subsequent analyses can be carried out with MutationalPatterns. Mutational signatures can be visualized with “plot_96_profile”, and the contribution of each signature in each sample can be visualized in a barplot with “plot_contribution”, in either the “absolute” mode, where the estimated total number of mutations is plotted per mutational signature, or in “relative” mode, where the same data are visualized as fractions (Fig. 1c). Alternatively, the signature contribution can be visualized in a heatmap with “plot_contribution_heatmap” (Fig. 2a), which also offers the possibility to hierarchically cluster samples based on Euclidean distance.

### Finding the contribution of known signatures in mutation catalogues

In addition to *de novo* signature extraction, the contribution of any set of signatures to the mutational profile of a sample can be quantified. This unique feature is specifically useful for mutational signature analyses of small cohorts or individual samples, as well as for relating new mutation data to known signatures and published findings. The non-negative linear combination of a set of user-specified mutational signatures that best reconstructs the mutation profile of a single sample can be determined by minimizing the Euclidean norm of the residual, i.e.:

$$ \underset{x}{\min}\parallel S\bullet x-d{\parallel}_2^2,\mathrm{where}\ x\ge 0 $$

Here, *S* is the signature matrix, *x* the signature weight (contribution) vector and *d* the original 96 mutation count vector for a sample. This problem can be considered as a non-negative least-squares (NNLS) optimization problem, which is a constrained version of the least-squares problem where the weights are not allowed to become negative. The NNLS problem is well studied, and a widely used algorithm for solving this problem is an active set method [24]. MutationalPatterns uses an R implementation of this algorithm from the *pracma* package (https://CRAN.R-project.org/package=pracma) in “fit_to_signatures”.

### Mutational profile similarity

To determine the similarity *α* between two mutational profiles *A* and *B*, each defined as a non-negative vector with *n* mutation types, the cosine similarity is calculated:

$$ \mathrm{sim}\left(A,B\right)=\alpha =\frac{\sum_{i=1}^n{A}_i{B}_i}{\sqrt{\sum_{i=1}^n{A}_i^2}\sqrt{\sum_{i=1}^n{B}_i^2}} $$

The cosine similarity can be calculated with “cos_sim” and has a value between 0 and 1. Two mutational profiles are identical when the cosine similarity is 1, and independent when the cosine similarity is 0. Because the cosine similarity evaluates the direction of the vectors and not the magnitude, it is not required to normalize the mutation profiles for the total number of mutations in a given sample.

### Mutational strand asymmetries

The involvement of transcription-coupled repair can be evaluated by testing for a transcriptional strand bias for the mutations that are located within gene bodies. While we cannot determine on which strand the original DNA damage occurred, we can regard the base substitutions from a reference frame of C > X or T > X changes (where X is any other base) and determine whether the mutated “C” or “T” base is located on the transcribed or non-transcribed strand. Since the gene definitions report the coding strand, which is untranscribed, base substitutions located on the same strand as the gene definitions are defined as “untranscribed” and on the opposite strand as “transcribed”. Gene definitions for each reference genome can be retrieved from the University of California, Santa Cruz (UCSC) Genome Browser [25] or BiomaRt [19] by loading a TxDb annotation package from Bioconductor. Subsequently, the transcriptional strand of all mutations within gene bodies can be determined with “mut_strand”.

**Fig. 3:**
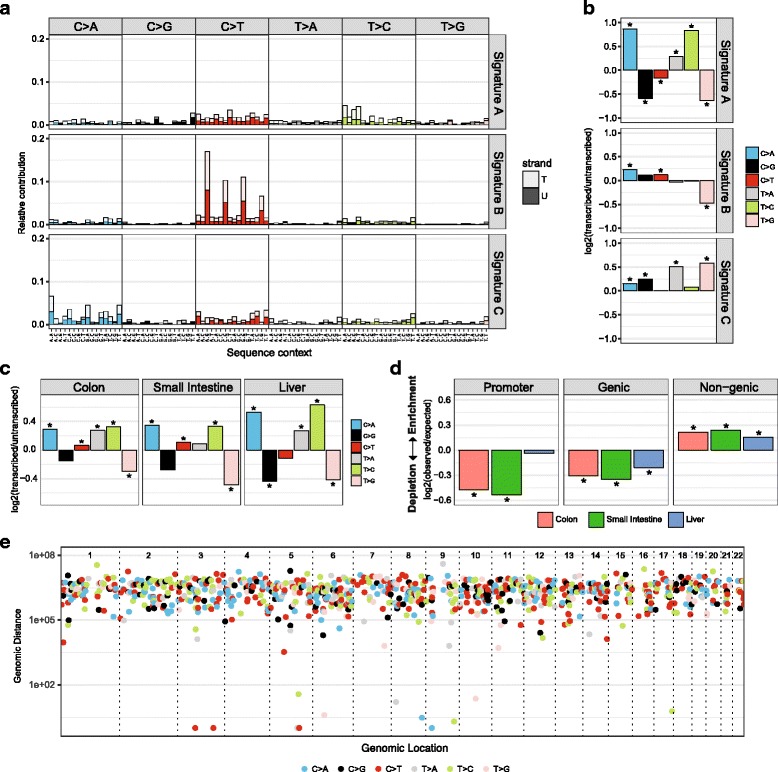
Transcriptional strand bias and genomic distribution. **a** Mutational signatures with transcriptional strand information. The relative contribution of each trinucleotide change, subdivided into the fraction of trinucleotide changes present on the transcribed (*T*, *light shades*) and untranscribed strand (*U*, *dark shades*) **b** Log2 ratio of the number of mutations on the transcribed and untranscribed strand per indicated base substitution for each signature depicted in **a**. The log2 ratio indicates the effect size of the bias and *asterisks* indicate significant transcriptional strand asymmetries (*P* < 0.05, two-sided binomial test). **c** Log2 ratio of the number of mutations on the transcribed and untranscribed strand per indicated base substitution for each tissue type. *Asterisks* indicate significant transcriptional strand asymmetries (*P* < 0.05, two-sided Poisson test) **d** Enrichment and depletion of somatic point mutations in the promoter regions, gene bodies and intergenic genomic regions for all tissues. The log2 ratio of the number of observed and expected point mutations indicates the effect size of the enrichment or depletion in each region. *Asterisks* indicate significant enrichments or depletions (*P* < 0.05, one-sided binomial test). **e** Rainfall plot showing the genomic location of mutations, intermutation distance and the mutation types for sample 14-b

The strand bias can be visualized for each sample with “plot_strand”, where the log2 ratio of the number of mutations on the transcribed and the untranscribed strand is used as the effect size of the strand bias. A Poisson test can be performed to assess the statistical significance of the strand bias using “strand_bias_test” (Fig. 3c). In addition, the involvement of replication-associated mechanisms can be evaluated by testing for a mutational bias between the leading and the lagging strand. The replication strand is dependent on the locations of replication origins from which DNA replication is fired. Replication timing is, however, dynamic and cell-type specific, which makes replication strand determination less straightforward. Replication timing profiles can be generated with Repli-Seq experiments [26]. Alternatively, replication timing datasets of human cell lines from the ENCODE project [27] are publicly available via the UCSC Genome Browser [25] and capture the conserved replication patterns. From replication timing profiles, the replication direction can be determined as described in [11]. Once the replication direction is defined, a strand asymmetry analysis can be performed using the same functions as for the transcription strand bias analysis. A replication direction example data file is provided with the package.

The transcriptional or replicative strand information can be included as an additional feature in the mutational signature analysis. Mutation count matrices with 192 features (96 trinucleotide changes * 2 strands) can be created with “mut_matrix_stranded”. Subsequently, mutational signatures with 192 features can be extracted with “extract_signatures”, and their profile visualized as a stacked barplot with “plot_192_profile”. The effect size and the statistical significance of the strand bias of the signatures can be visualized with “plot_signature_strand_bias” (Fig. 3b).

### Genomic distribution

To determine whether base substitutions appear more or less frequently in specific genomic regions, the ratio of the observed and expected mutations in the genomic regions is determined with “genomic_distribution”. For this analysis, the chance of observing a mutation at one base is calculated as the total number of mutations that were identified in a sample, divided by the total number of bases in the genome that were surveyed in that sample with the sequencing experiment. Subsequently, the resulting overall mutation rate is multiplied by the length of the genomic region that is surveyed in that sample, to calculate the expected number of mutations in that genomic region. The “surveyed” bases are positions in the genome at which there are enough high-quality reads to reliably call a mutation in that sample, and can be determined using the CallableLoci tool of the Genome Analysis Toolkit (GATK) [28]. A list with GRanges of regions that were surveyed for each sample should be passed to “genomic_distribution”. If a surveyed area would not be included in this analysis, it might result in e.g. a depletion of mutations in a certain genomic region that is solely a result from a low coverage in that region and therefore does not represent an actual depletion of mutations.

The statistical significance of the enrichment or depletion is calculated with a one-sided binomial test with “enrichment_depletion_test”. This test can be performed per sample, or per sample group, which can be specified using the “by” parameter. Subsequently, the enrichment or depletion can be visualized with “plot_enrichment_depletion” (Fig. 3d). All genomic regions can be tested as long as they are represented as GRanges objects [20]. The genomic regions can be based on experimental data or publicly available annotation data retrieved via e.g. BiomaRt [19], such as promoters, CTCF binding sites and transcription factor binding sites. Finally, a rainfall plot can be made with “plot_rainfall” (Fig. 3e) to visualize the intermutation distance and mutation types and identify the localized hypermutation termed “kataegis”.

## Results and discussion

We illustrate MutationalPatterns using somatic mutation catalogues of 45 human adult stem cells (ASCs) of three different tissues: liver, small intestine and colon [29]. The spectrum of base substitution types reveals a different mutational landscape for liver ASCs compared with intestinal ASCs (Fig. 1a), illustrating that this analysis can be used to detect gross differences in the activity of mutational processes. Deeper investigation into the processes can be achieved by performing a *de novo* extraction of mutational signatures using NMF.

We extracted three mutational signatures (Fig. 1b). Signature B has a high contribution in intestinal ASCs specifically (Fig. 1c). We calculated the similarity of these signatures with COSMIC mutational signatures using “cos_sim_matrix”. Signature B is highly similar to COSMIC S1 (α = 0.99, Fig. 1d), which is attributed to spontaneous deamination of methylated cytosines at CpG sites [5]. In liver ASCs, Signature A shows the largest contribution, which was found to be similar to both S5 and S16 (α = 0.93 and 0.88 respectively, Fig. 1d). The underlying molecular mechanisms of these signatures are unknown, but both signatures are reported to have a transcriptional strand bias (http://cancer.sanger.ac.uk/cosmic/signatures). Consistently, transcriptional strand bias analysis of the mutation catalogues detects a strong bias for Signature A (Fig. 3a, b), confirming the likely involvement of transcription-associated molecular mechanisms [11]. Lastly, Signature C is most similar to COSMIC signature 18 (α = 0.83, Fig. 1d), of which the aetiology is currently unknown.

While the *de novo* signature extraction is a very powerful method for the identification of new signatures, it has several disadvantages. The analysis requires mutation sets with a large number of samples with diverse mutation spectra, as it relies on the dimensionality reduction method NMF. In order to evaluate the presence of the signatures in an additional sample, it must be added to the existing dataset and the complete analysis should be executed again. As a result, the input matrix will grow, and the runtime will increase with *O(n*^*3*^*)*, where *n* is the number of samples, which makes this approach computationally demanding. Moreover, the extracted mutational signatures will slightly change every time a new sample is added.

Alternatively, the contribution of previously identified mutational signatures can be quantified in a single sample with the “fit_to_signatures” feature of MutationalPatterns. To demonstrate the ability of the “fit_to_signatures” to reliably estimate signature contributions, we re-estimated the contribution of the three signatures that were *de novo* extracted with NMF (Fig. 1b), in the samples using “fit_to_signatures”. We find that the signature contribution is very similar between the two methods (average Pearson correlation = 0.98, Additional file 1: Figure S1). Furthermore, the analysis is very fast with a runtime of approximately 0.1 s for 45 ASC samples (Additional file 1: Figure S2C), and is scalable with *O(n)*, where *n* is the number of samples. Unlike NMF, the result of this analysis is independent of other samples. This functionality can be used to study the activity of previously identified mutational signatures in cells with altered DNA damage or repair, which will help to uncover the molecular process underlying the mutational signature. Moreover, this type of analysis is useful for clinical applications, as it allows for a fast per-patient analysis of the contribution of known signatures to their mutation profile.

By fitting the ASC mutational profiles to COSMIC signatures, we find that the mutational landscape of intestinal ASCs can be predominantly reconstructed with a high contribution of S1, and liver ASCs with S5 and S16 (Fig. 2a). In line with this, the *de novo* extracted signatures show a high similarity to these COSMIC signatures (Fig. 1d). However, not all COSMIC signatures that are similar to the *de novo* extracted signatures are required to reconstruct a mutational profile. This is because COSMIC mutational signatures are not independent; some have high cosine similarities (Additional file 1: Figure S3). For example, S1 and S6 are similar (α = 0.84), and correspondingly the de novo extracted Signature B is similar to both S1 and S6 (Fig. 1d). However, to reconstruct the intestinal mutational profiles, only S1 is required (Fig. 2a).

To test how well each mutational profile can be explained by the provided mutational signatures, the cosine similarity can be calculated between the original and the reconstructed mutational profile. The mutational profiles of most ASCs can be reconstructed very well with the COSMIC signatures (mean α = 0.98, Fig. 2b), while some ASCs are not fully reconstructed (α < 0.95, Fig. 2b). This check is important, as a low similarity between the original and the reconstructed profile indicates that the analysed mutational profile cannot be fully explained by the provided signatures, which suggests that additional, unassessed mutational processes might underlie the observed catalogue of somatic mutations. Comparison of the original with the reconstructed mutational profile reveals which trinucleotide peaks cannot be reconstructed with the given signatures and provides important leads on the missing mutational mechanisms active in the system studied (Fig. 2c).

**Fig. 4:**
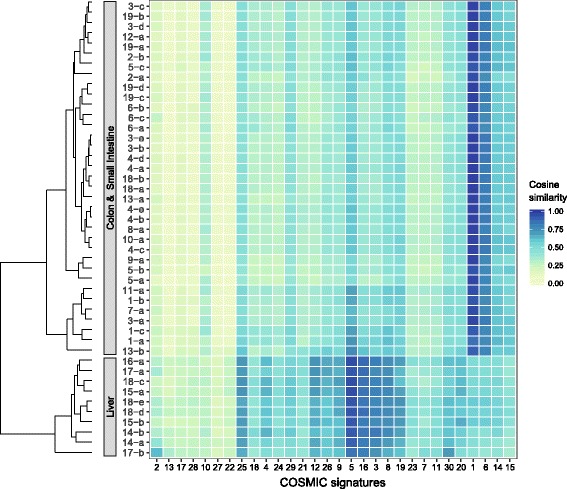
Heatmap of cosine similarities between the mutational profile of each sample and COSMIC signature. The samples are hierarchically clustered (average linkage) using the Euclidean distance between the vectors of cosine similarities with the signatures. The signatures have been ordered according to hierarchical clustering (average linkage) using the cosine similarity between signatures, such that similar signatures are displayed close together

Next, we determined the similarity between each mutational profile and each COSMIC signature, which reflects how well each mutational profile can be explained by each signature individually. We visualized these similarities in a heatmap using “plot_cosine_heatmap” (Fig. 4). COSMIC signatures that have a very similar profile, such as S5 and S16 (α = 0.9, Additional file 1: Figure S3), will have comparable cosine similarity values (Fig. 4). The advantage of this cosine heatmap representation is that it shows at a glance the similarity in mutation profiles between samples, while at the same time providing information on which signatures are likely active. Hierarchical clustering of the samples using the Euclidean distance between their cosine similarity values clearly separates the liver ASCs from the intestinal ASCs, while the colon and the small intestinal ASCs are not distinguishable by tissue-specific profiles (Fig. 4). This analysis demonstrates the utility of the MutationalPatterns package to detect and visualize sample groups with a similar activity of mutational processes.

Finally, we evaluated the enrichment and depletion of mutations in promoters, genes and non-genic regions. We downloaded these genomic annotations using BiomaRt [19]. Intestinal ASCs show a depletion of mutations in promoter regions, whereas liver ASCs do not (Fig. 3d). This lack of depletion could be explained by binding of transcription factors to promoters, which can impair NER and result in increased rate of mutations at active promoters [30, 31]. Furthermore, all ASC types show a depletion of mutations in genes and an enrichment in non-genic regions. This is expected, as genes are typically located in early-replicating genomic regions, where the activity of MMR is known to be higher than in late-replicating regions [32]. In addition, expressed genomic regions may benefit from the presence of DNA damage repair through TC-NER and/or transcription domain-associated repair (DAR) [33, 34]. The mutations in liver ASCs show the strongest transcriptional strand bias (Fig. 3c), indicating a high activity of TC-NER in these relatively quiescent cells. Nevertheless, the depletion in genes is larger in the intestinal ASCs compared with liver ASCs (Fig. 3d), which may indicate that either replication-associated repair or DAR is more active in the highly proliferative intestinal ASCs. These results illustrate that the genomic distribution analysis provides important clues on the underlying mutational processes. Further assessment of the involvement of DNA repair mechanisms can be achieved by performing mutational signature and strand bias analyses per genomic region, which is possible when there are sufficient mutations located in the genomic regions.

### Comparing methods

An overview of the functionalities of MutationalPatterns and related software tools can be found in Table 1.

**Table 1:** MutationalPatterns feature overview and comparison with related software tools

Functionality	Analysis	Mutational patterns	pmsignature [23]	MutSpec [36]	Somatic Signatures [37]	deconstructSigs [35]	EMu [38]
	Language/platform	R	R	Galaxy	R	R	C++
Mutational characteristics	Mutation spectrum	X	X	X	X	X	–
	Transcriptional strand bias	X	–	X	–	–	–
	96 mutation profile	X	–	X	X	–	X
Mutational signatures	Signature extraction (NMF)	X	X	X	X	–	–
	Signature extraction (NMF) with strand bias	X	X	–	–	–	–
	Signature contribution heatmap	X	–	–	X	–	–
	Signature contribution barplot	X	–	X	X	–	X
	Hierarchical sample clustering based on signature contribution	X	–	X	X	–	–
	Signature similarity heatmap	X	–	X	–	–	–
	Plot and compare two 96 profiles	X	–	–	–	X	–
	Sample signature similarity heatmap	X	–	–	–	–	–
	Find optimal linear combination of known signatures	X	–	–	–	X	–
Genomic distribution	Rainfall plot/mutation clustering along the genome	X	–	–	–	–	X
	Enrichment/depletion in genomic regions	X	–	–	–	–	X

An important advantage of MutationalPatterns over other available software tools is that it brings together many informative pattern analyses in a single package. Because MutationalPatterns is implemented within the R/Bioconductor platform, it integrates with common R genomic analysis workflows and allows easy association with publicly available annotation data. Moreover, MutationalPatterns can be used to easily generate publication-ready visualizations, while maintaining lay-out flexibility. The functionality to determine the activity of mutational processes through signature analyses in a single sample is an important feature. To date, only *deconstructSigs* provides this functionality, which also minimizes the RSS between the original and reconstructed mutational profile. The deconstructSigs package uses a heuristic approach with ad hoc thresholds to solve this optimization [35], while MutationalPatterns uses a fast implementation of the general and theoretically well-founded NNLS algorithm by Lawson and Hanson (https://CRAN.R-project.org/package=pracma). We compared the performance of the “fit_to_signatures*”* function of MutationalPatterns with the “whichSignatures*”* of deconstructSigs. We used both functions to find the optimal linear combination of 30 COSMIC mutational signatures to reconstruct the somatic mutation profiles of the 45 human ASCs, starting from a mutation count matrix. The linear combinations of mutational signatures that were determined by these packages were highly similar (average Pearson correlation = 0.98, Additional file 1: Figure S2A). We reconstructed the mutation profiles using the obtained signature weights and compared them with the original mutation profiles. The similarities and discrepancies between the original and reconstructed mutation profiles were comparable for MutationalPatterns (mean α = 0.978, mean RSS = 1.38e-03) and deconstructSigs (mean α = 0.977, mean RSS = 1.40e-03). Importantly, the MutationalPatterns analysis runtime is approximately 400 times faster compared with deconstructSigs (Additional file 1: Figure S2C).

## Conclusions

MutationalPatterns is a flexible and comprehensive R/Bioconductor package that allows researchers to rapidly assess a wide range of mutation characteristics in catalogues of somatic base substitutions. We showed that by analysing such patterns in concert, valuable clues on the molecular mechanisms underlying mutation accumulation can be revealed. MutationalPatterns allows researchers to generate publication-ready visualizations, which can be easily adapted to individual requirements.

In the past few years, mutational signature analyses have gained much interest, and some have been shown to have diagnostic value [6, 8]. Since the aetiology of most identified signatures is currently unknown, deeper investigation into the underlying molecular mechanisms will be essential to unfold signature analysis to its full potential. MutationalPatterns provides a very efficient method to determine the contribution of known mutational signatures in single samples, without requiring large datasets. This functionality will allow researchers to molecularly dissect well-established mutational signatures, by studying their contribution in cells with altered DNA damage or repair.

Finally, we anticipate that the ability to determine the activity of mutational signatures within individual patient samples has the potential to reveal molecular perturbations and thereby improve both diagnosis and treatment strategies. Furthermore, this analysis can facilitate novel biomarker discovery by associating mutational signature activity with treatment response. Taken together, we anticipate that MutationalPatterns will support fundamental research into mutational mechanisms, as well as enhance the knowledge that can be retrieved from individual patient sequencing data.

## Availability and requirements

The availability and requirements are listed as follows:

Project name: MutationalPatterns

Project home page: https://github.com/UMCUGenetics/MutationalPatterns

Archived version: https://bioconductor.org/packages/3.6/bioc/html/MutationalPatterns.html

Operating system(s): Linux, Windows or MacOS

Programming language: R (version > = 3.4.0)

License: MIT

## Additional file

Additional file 1: **Figure S1.** Signature contributions as estimated by NMF and NNLS. **Figure S2.** Comparison between MutationalPatterns (fit_to_signatures) and deconstructSigs (whichSignatures). **Figure S3.** COSMIC signature similarities. (PDF 3146 kb)

## List of abbreviations

ASC: Adult Stem Cell
BER: Base excision repair
COSMIC: Catalogue of Somatic Mutations in Cancer
HR: Homologous Recombination
MMR: Mismatch repair
NER: Nucleotide Excision Repair
NMF: Non-negative Matrix Factorization
NNLS: Non-Negative Least Squares
PARP: Poly (ADP-Ribose) Polymerase
RSS: Residual Sum of Squares
TC-NER: Transcription-Coupled Nucleotide Excision Repair
VCF: Variant Call Format
WGS: Whole Genome Sequencing
