# Supplementary material for: MutationalPatterns: comprehensive genome-wide analysis of mutational processes
Source: Genome Med. 2018 Apr 25;10:33. doi: 10.1186/s13073-018-0539-0 (PMC5922316; doi:10.1186/s13073-018-0539-0)
Supplement: Supplementary file 1 — Figure S1. Signature contributions as estimated by NMF and NNLS. Figure S2. Comparison between MutationalPatterns (fit_to_signatures) and deconstructSigs (whichSignatures). Figure S3. COSMIC signature similarities. (PDF 3146 kb) [file 13073_2018_539_MOESM1_ESM.pdf]

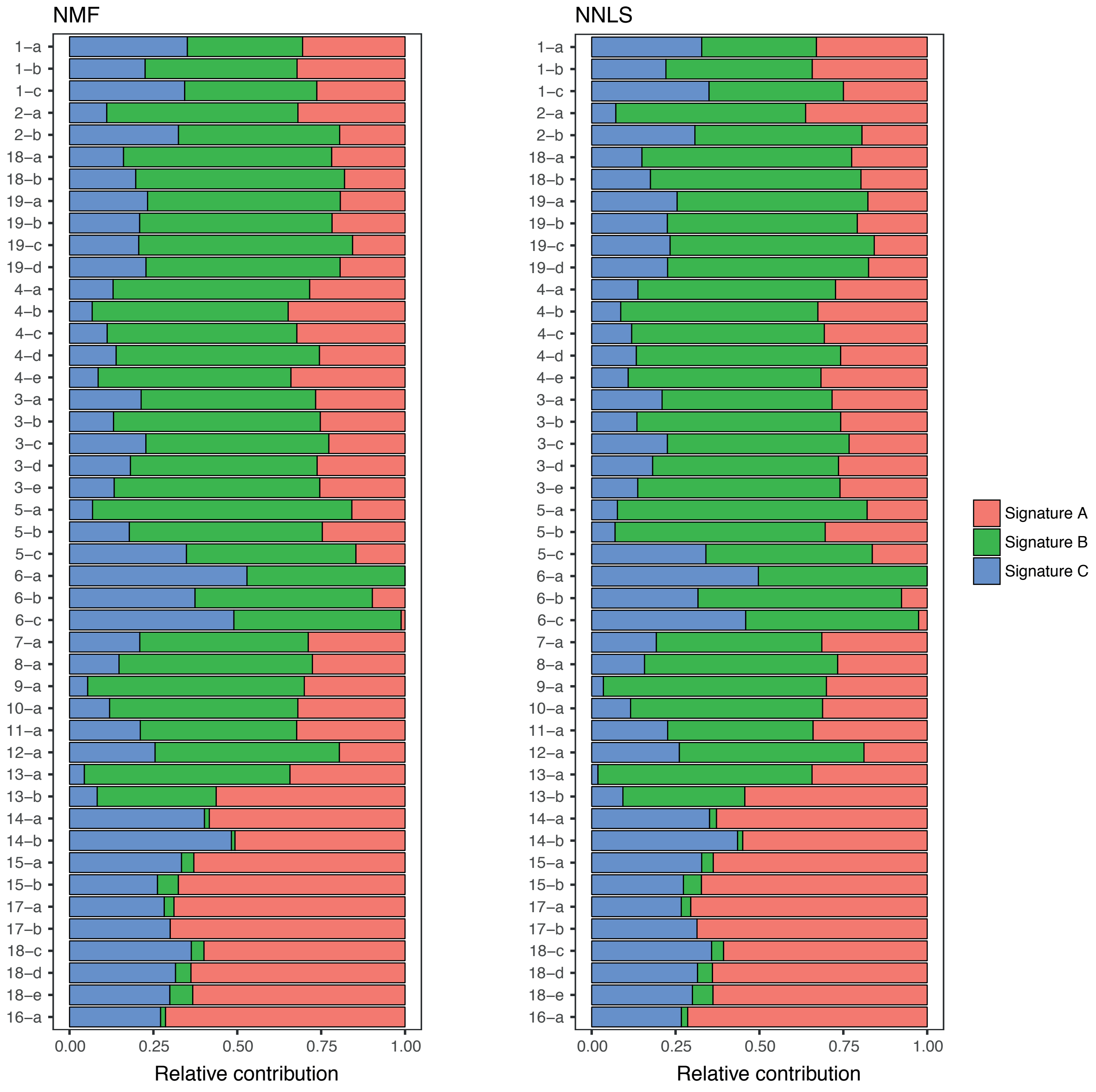

Fig. S1 Signature contributions as estimated by NMF and NNLS. Relative contribution of the three indicated mutational signatures (depicted in Fig. 1B) for each sample as estimated by NMF (left) and NNLS (right).

A

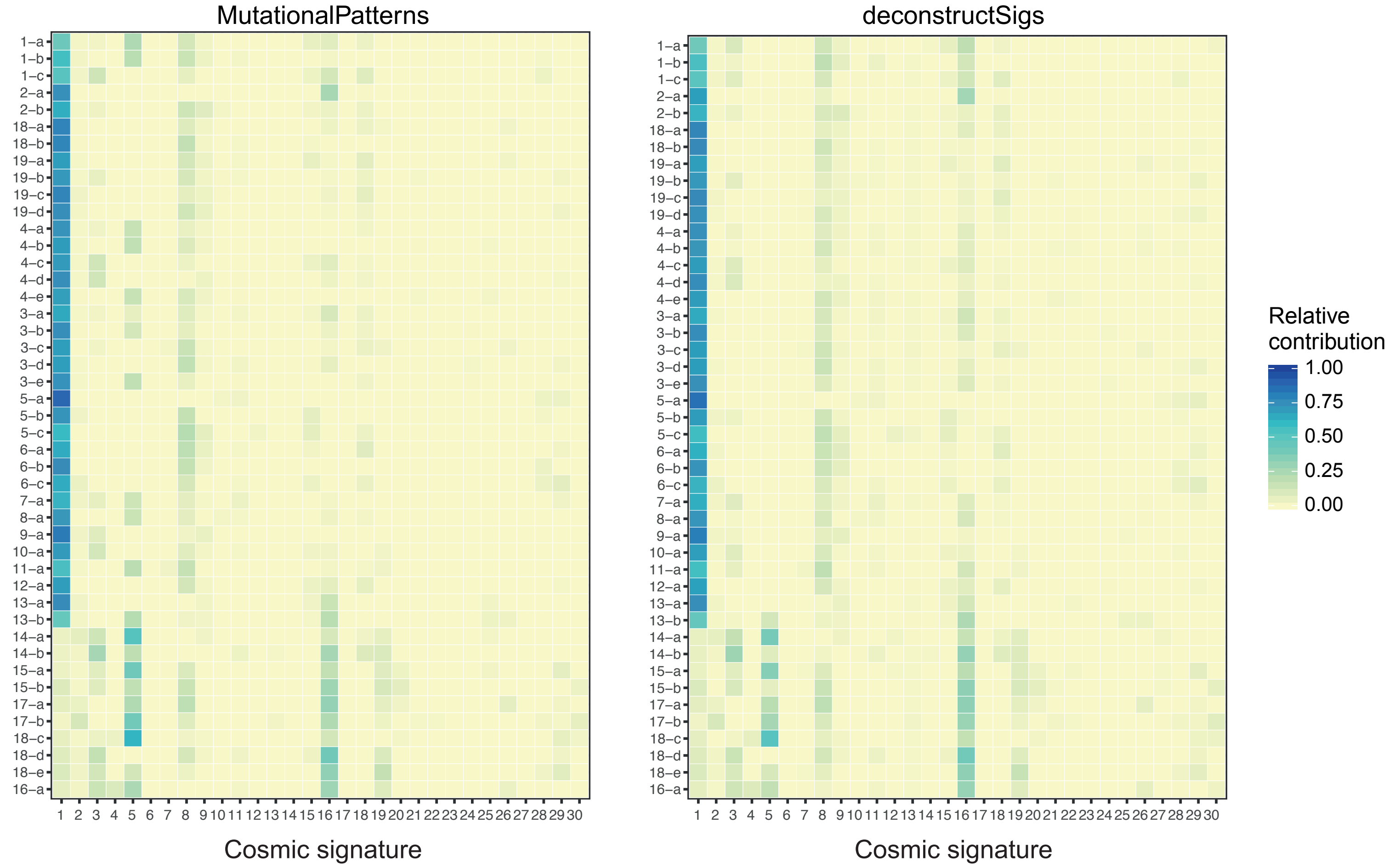

B

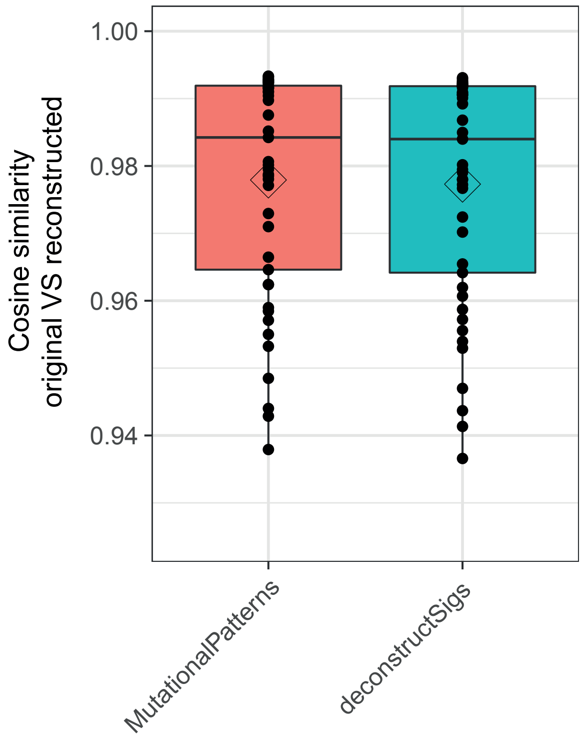

C

COSMIC signatures similarity heatmap.

	MutationalPatterns	deconstructSigs
Runtime (45 VCFs)	0.1 s	42.5 s

Fig. S2 Comparison between MutationalPatterns (fit_to_signatures) and deconstructSigs (whichSignatures). a. Relative contribution of all 30 COSMIC signatures for each sample, as found by MutationalPatterns (left) and deconstructSigs (right). **b.** Cosine similarity between the original and the reconstructed mutational profiles for MutationalPatterns and deconstructSigs. **c.** The runtime (elapsed time) in seconds to find the optimal linear combination of mutational signatures for 45 somatic mutation catalogs for both packages; MutationalPatterns is approximately 400 times faster than deconstructSigs.

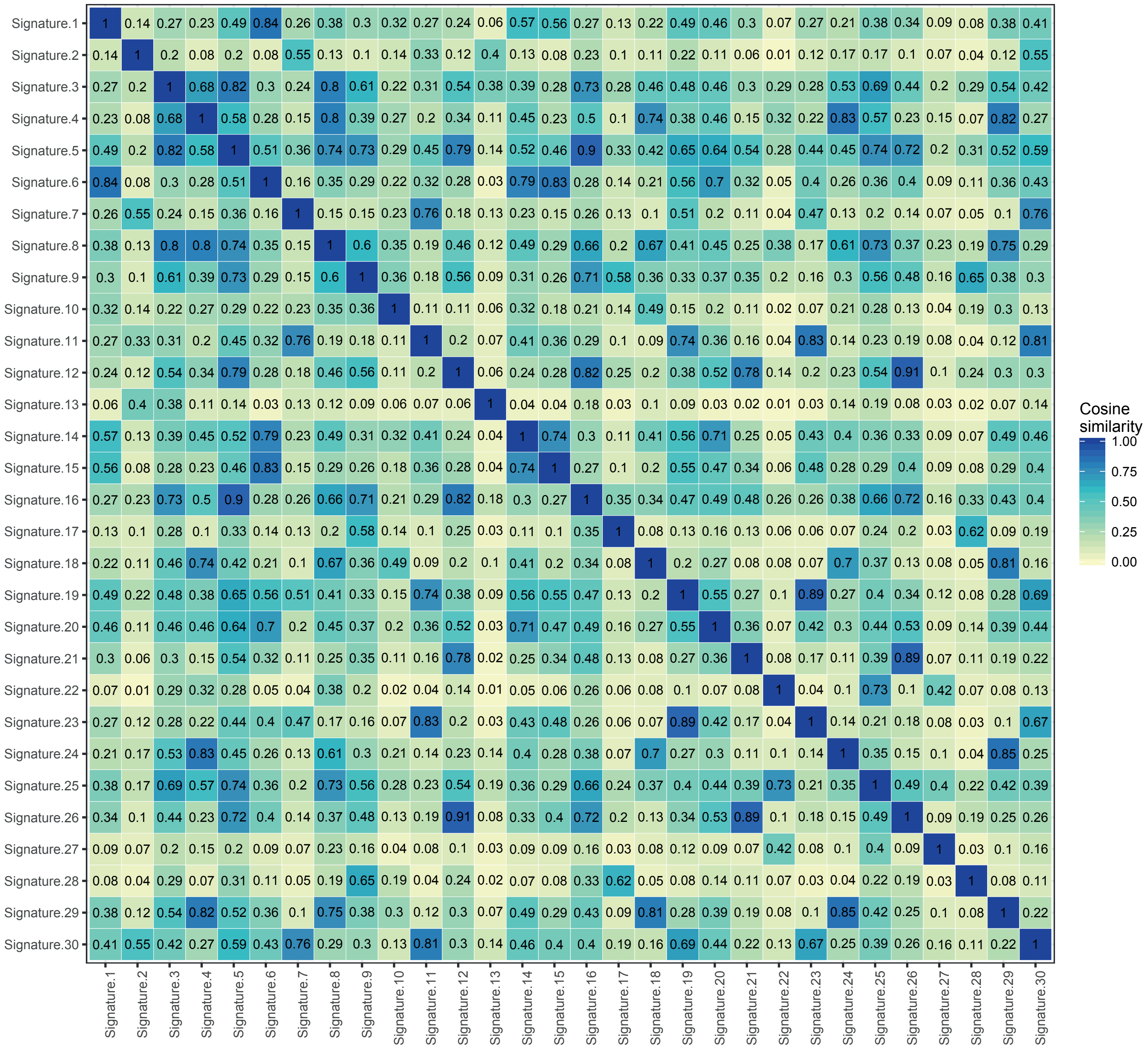

Fig. S3 COSMIC signature similarities. Heatmap with cosine similarities between all 30 COSMIC mutational signatures.
